# Lumbar puncture use and antibiotic treatment in newly admitted febrile children following index cases of invasive infections

**DOI:** 10.1186/s12879-026-13486-1

**Published:** 2026-05-20

**Authors:** Maximilian David Mauritz, Mathias Sehlbrede, Julia Wager, Malik Aydin

**Affiliations:** 1 Chair of Pediatrics, Children’s and Adolescents’ Hospital Datteln, Witten/Herdecke University, Datteln, Germany; 2 Department of Children’s Pain Therapy and Pediatric Palliative Care, Faculty of Health, School of Medicine, Witten/Herdecke University, Witten, Germany; 3 PedScience Research Institute, Datteln, Germany; 4 German Pediatric Pain Center, Children’s and Adolescents’ Hospital Datteln, Witten/Herdecke University, Datteln, Germany; 5 Laboratory of Translational Medicine and Pediatric Infectious Diseases, Center for Biomedical Education and Science (ZBAF), Department of Human Medicine, Faculty of Health, Witten/Herdecke University, Witten, Germany

**Keywords:** Sepsis, Meningococcal sepsis, Bacterial meningitis, Infection, Antibiotic treatment, Antimicrobial stewardship

## Abstract

**Background:**

Bacterial meningitis (BM) and meningococcal sepsis are rare but serious infectious events in childhood. Despite the availability of validated decision tools and guidelines, clinical practice may be influenced by subjective risk perception, in particular after rare, high-impact events. This study investigated whether in-hospital cases of BM or meningococcal sepsis were associated with changes in lumbar puncture (LP) frequency or empirical antibiotic use among subsequently evaluated febrile infants and children.

**Methods:**

We performed a retrospective single-center study of pediatric inpatients (29 days to 18 years) undergoing LP between January 2019 and December 2024. Clinical, microbiological, and treatment data were analyzed, and the Bacterial Meningitis Score (BMS) was calculated. Multilevel logistic regression models assessed associations between events of BM or meningococcal sepsis, LP frequency, and antibiotic treatment for suspected BM.

**Results:**

Of 30,967 inpatients, 269 LPs were performed for suspected BM. Five cases of BM were identified, viral pathogens were detected in 24 patients. LP frequency increased significantly in a 4-month period following a BM or meningococcal sepsis case that occurred at the hospital (OR = 2.06, 95% 0.96, 4.45), but not in subsequent months. There was no corresponding increase in antibiotic therapy observed. Both younger age and non-negative BMS were associated with receiving BM-targeted antibiotics.

**Conclusions:**

Rare invasive infectious events were associated with a short-term rise in LP use but not with increased antibiotic treatment, suggesting a transient shift in diagnostic behavior. These findings highlight the need to address cognitive influences on clinical decision-making and to reinforce structured diagnostic pathways and antimicrobial stewardship.

**Clinical trial number:**

Not applicable.

**Supplementary Information:**

The online version contains supplementary material available at 10.1186/s12879-026-13486-1.

## Introduction

Invasive bacterial infections, such as bacterial sepsis or bacterial meningitis (BM), are rare but serious infectious diseases associated with high morbidity and mortality, particularly in infants and young children [1]. Due to the potentially fulminant course of the disease, rapid diagnosis and therapy represent a major challenge for clinicians [2]. In evaluating febrile infants, invasive diagnostic measures include obtaining blood for inflammatory biomarker testing and microbiologic cultures. In cases of children who appear ill, elevated infection parameters, or neurological symptoms, a lumbar puncture (LP) for cerebrospinal fluid (CSF) analysis should be performed to diagnose BM [3]. In sick children, the early initiation of antibiotic therapy is a core element in daily practice for treating severe bacterial infections.

On the other hand, the indications for such measures and treatments should be clearly defined due to rare but potentially serious complications such as infection, spinal bleeding, or post-LP pain [4]. This also includes the possible complications and discomfort associated with hospitalization and parenteral antibiotic treatment [5]. Similarly, the typical broad empirical antimicrobial therapy for these infections should only be administered to children who are potentially ill. When there is no evidence of disease, antimicrobial therapy should not be initiated to avoid selecting for resistant bacteria and to avoid repeated painful venipunctures. Treatment that has already started should be discontinued soon once a bacterial infection has been completely ruled out [6].

The decision to perform invasive diagnostic testing such as a LP or to start empirical antibiotic treatment is usually based on a combination of the patient’s appearance, (preliminary) laboratory results, and the clinician’s judgement [7]. In clinical practice, validated scores, such as the “Bacterial Meningitis Score” (BMS) [8], a clinical decision tool to assess the risk of BM, as well as evidence-based guidelines, support rational decisions for diagnosis and therapy. However, personal experiences of severe courses of bacterial infections in the clinical setting psychologically affect medical decisions [9]. Thus, fear of very rare courses of sepsis [10] or BM [11] might cause overdiagnosis and overtreatment. For example, the threshold for invasive diagnostics or prescribing broad-spectrum antibiotics could be lower. So far, there is limited knowledge regarding these phenomena in the field of pediatrics.

Therefore, our study aims to investigate the relationship between events of meningococcal sepsis or BM related to subsequent LP procedures and antibiotic treatments in newly admitted febrile children at a university pediatric hospital. Results may enable hypothesis-building about potential psychological factors influencing decision-making in clinical practice and, if necessary, the development of strategies to support evidence-based decision-making.

## Materials and methods

### Study design

Medical records and routine microbiological diagnostic data were extracted from the hospital information system for pediatric inpatients treated at a tertiary care pediatric hospital of Witten/Herdecke University, Germany, between January 1, 2019, and December 31, 2024.

### Data collection

The incidence of LP was counted using the German operation and procedure classification system (OPS-2024), Code 1-204.2 (LP for cerebrospinal fluid collection). Subsequent data extraction included examination of preliminary diagnosis upon admission or referral to the hospital, the indication for CSF collection, the incidence and duration of antibiotic therapy, laboratory data regarding BM, and final diagnoses upon discharge from the hospital. The BMS is a validated clinical prediction rule for identifying children beyond the neonatal period with CSF pleocytosis and a very low risk of BM [8], retrospectively calculated from clinical and laboratory data. A negative BMS indicates that the risk of bacterial meningitis is extremely low and can be ruled out with very high sensitivity and negative predictive value [12]. Microbiological results for bacterial and viral pathogens in blood and CSF cultures were obtained through the hospital information system. In addition, the occupancy rates of the evaluated acute care, non-surgical hospital wards were analyzed for the period under review. The study recorded the time points when microbiologically confirmed BM or meningococcal sepsis occurred as “events.”

Inclusion criteria included all pediatric patients aged ≥ 29 days to < 18 years who received inpatient care during the study period (January 1, 2019, to December 31, 2024). The analysis included all patients who underwent LP during their hospital stay. The exclusion criteria include patients undergoing elective LP for non-infectious neurological or oncological conditions, as well as patients under ≤ 28 days of age.

### Statistical analysis

The statistical analysis was performed using IBM SPSS Statistics (Version 31.0, Armonk, NY, USA) and R 4.5.1 (R Core Team, Vienna, Austria). A multilevel logistic regression model (R package lme4 [13]) with lmerTest [14] was used to test the research questions. At the cluster level, the year was the grouping variable (persons clustered within years). All models contained only random intercepts. For the model testing the association with undergoing a lumbar puncture (0 = no LP; 1 = LP), the time and event were used as predictor variables. Any BM or meningococcal sepsis event was binary-coded as 0 = no event, 1 = event, while the time-predictor variables cover each month of a year. The model used to test the association with BM treatment (0 = no treatment; 1 = treatment) includes age, centered at the grand mean, and a binary BMS variable (0 = BMS > 0; 1 = BMS = 0) as predictors. Significant interaction effects were analyzed with the Johnson-Neyman technique in the R package interactions [15]. We compared therapy duration across age groups (0–2, 2–6, and 6–18 years) using a Welch one-way ANOVA. All analyses, along with the full code, are available in Supplementary material 1.

### Ethics

The study was conducted in accordance with the principles outlined in the Declaration of Helsinki and its later amendments, and was approved by the Ethics Committee of Witten/Herdecke University, Germany (ID S-02/2025). This project involved no external funding or commercial support.

## Results

**Fig. 1:**
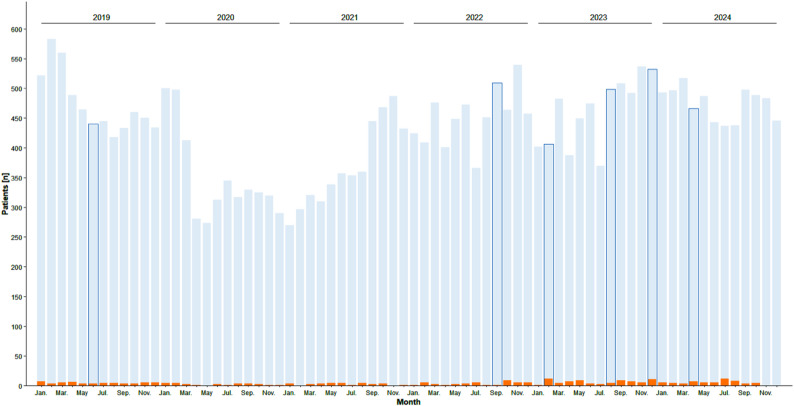
Monthly occupancy figures for the pediatric wards examined during the five-year study period (light blue) and monthly lumbar punctures (orange). Months with bacterial meningitis or meningococcal sepsis are highlighted in dark blue

Overall, *n* = 1,414 LPs were performed from January 1, 2019, to December 31, 2024. 1,145 LPs were excluded from further analysis because they were performed as elective LPs for oncological (*n* = 535), non-infectious neurological (*n* = 405), other conditions (*n* = 83), facial nerve palsy (*n* = 21) or because the patients were under the age of 29 days (*n* = 101). The remaining 269 LPs were included in the analysis. A total of 30,967 inpatients were treated in the acute care wards of the hospital during this period. Monthly occupancy figures for the acute care, non-surgical pediatric wards examined over the five-year study period, along with monthly lumbar punctures, are shown in Fig. 1.

### Patient characteristics

The mean age (standard deviation, SD) of patients undergoing LP was 2.9 years (± 4.25), ranging from 0 to 17.7 years. 47.9% of patients were younger than one year. The patient population consisted of *n* = 159 (59.1%) male and *n* = 110 (40.9%) female patients. The BMS was negative in 45% of cases (*n =* 121), BMS = 1 in 45.7% of cases (*n =* 123), BMS = 2 in 8.2% of cases (*n =* 22), BMS = 3 in 0.7% of cases (*n =* 2), and BMS = 4 in 0.4% of cases (*n =* 1). Based on the clinical presentation or medical history described, BM was a possible differential diagnosis in 69.5% of cases. Clinical and treatment characteristics of the included patients are summarized in Table 1.

**Table 1:** Clinical characteristics of the included patients (*n* = 269)

Characteristic	Value
Female	110 (40.9%)
Male	159 (59.1%)
Age, years (mean ± SD, range)	2.9 ± 4.25 (0–17.7)
Age < 1 year	142 (52.79%)
Length of hospital stay, days (mean ± SD)	6.9 ± 9.3
BMS = 0	121 (45.0%)
BMS = 1	123 (45.7%)
BMS = 2	22 (8.2%)
BMS = 3	2 (0.7%)
BMS = 4	1 (0.4%)
**Suspected bacterial meningitis and antibiotic therapies**	
Suspected meningitis/encephalitis	187 (69.5%)
Any antibiotic therapy	143 (53.2%)
Duration of antibiotic therapy, days	3.9 ± 2.9
Duration for confirmed BM, days	11.4 ± 6.3
**Final diagnoses**	
Viral infection	45 (16.7%)
Fever of unknown origin	36 (13.4%)
Febrile seizures	35 (13.0%)
Viral meningitis	29 (10.8%)
Urinary tract infection	20 (7.4%)
Sepsis	17 (6.3%)
Gastroenteritis	16 (5.9%)
Pneumonia	11 (4.1%)
Encephalitis	9 (3.3%)
Bacterial meningitis	7 (2.6%)
Other diagnoses	43 (16.0%)

BM: bacterial meningitis; SD: standard deviation

### Pathogens in lumbar puncture and blood culture

A pathogen was detected in the CSF of 24 patients. These were primarily viral (enterovirus, *n =* 13; human herpesvirus 7, *n =* 3; human parechovirus, *n =* 3; parvovirus B19, *n =* 1). Bacterial pathogens were detected in the CSF in five cases, including *Streptococcus pneumoniae* (*n =* 2), *Staphylococcus aureus* (*n =* 2), and *Neisseria meningitidis* (*n =* 1). The patients’ ages were 0.2 and 9.4 years (*S. pneumoniae*); 0.8 and 12.2 years (*S. aureus*), and 0.3 years (*N. meningitidis*).

In six cases of suspected BM, bacterial pathogens were isolated from blood cultures: *N. meningitidis* (*n =* 3), *S. pneumoniae* (*n =* 1), *S. pyogenes* (*n =* 1), and *S. aureus* (*n =* 1). In one case, *N. meningitidis* was detected only in the blood culture.

Among patients with a negative BMS (BMS = 0), there was one case of BM (0.83%) and ten cases of viral detection in the CSF (8.3%). Among those with a BMS of 1, BM occurred in 2 cases (1.63%), and 8 cases had viral detection in the CSF (6.5%). In patients with a BMS of 2, BM was observed in one case (4.5%). In patients with BMS scores of 3 or 4, BM was identified in all cases. The single patient with detection of bacterial pathogens in the CSF despite a negative BMS had already been treated with antibiotics at the time of the LP, in which the child had been initially assessed as critically severe ill prior to the LP. All pathogens detected in CSF and blood cultures are summarized in Table 2.

**Table 2:** Pathogens detected in cerebrospinal fluid and blood cultures

Finding	*n* (%)	Under one year of age: *n* (% of total)
**Patients with pathogen in CSF**	24	14 (58,33%)
Enterovirus	13 (54.2%)	7 (29,2)
HHV-7	3 (12.5%)	0
Human parechovirus	3 (12.5%)	3 (12.5%)
Parvovirus B19	1 (4.2%)	1 (4.2%)
*S. pneumoniae*	2 (8.3%)	1 (4.2%)
*S. aureus*	2 (8.3%)	1 (4.2%)
*N. meningitidis*	1 (4.2%)	1 (4.2%)
**Bacterial pathogens in blood culture**	6	6 (100%)
*N. meningitidis*	3 (50%)	3 (50%)
*S. pneumoniae*	1 (16.7%)	1 (16.7%)
*S. pyogenes*	1 (16.7%)	1 (16.7%)
*S. aureus*	1 (16.7%)	1 (16.7%)

CSF: cerebrospinal fluid; HHV: human herpesvirus

### Treatment characteristics

The mean length of stay (SD) in hospital was 6.9 days (± 9.3), with a range of 1 to 155 days. 53.2% of patients received antibiotic treatment effective against BM. Furthermore, antibiotic therapies were administered for a mean (SD) of 3.9 (± 2.9) days. In detail, the antibiotic therapy consisted of cephalosporin plus ampicillin in 68.4% of cases (*n* = 99), cephalosporin alone in 28.3% (*n* = 41), and other therapeutic regimens in 3.5% (*n* = 5). In 89.1% of cases, antiviral therapy with acyclovir was used, and in 4.2% of cases, antibiotic combination therapy with gentamicin was used. In patients over one year of age, treatment consisted of cephalosporin in 54.4% of cases, cephalosporin plus ampicillin in 42.2% of cases, and other therapies in 3.6% of cases. Acyclovir was used in 91.3% of cases.

The mean (SD) duration of antibiotic treatment for BM was 11.4 (± 6.3) days. The maximum duration of therapy was 21 days. There were no statistically significant differences in treatment duration among patients aged 0–2 (*n* = 106), 2–6 (*n* = 17), and 6–18 years (*n* = 19), F(2, 31.41) = 0.08, *p* = 0.927, η² = 0.005. The final diagnoses for patients discharged after LP were distributed as follows: viral infection (16.7%), fever of unknown origin (13.4%), febrile seizures (13%), viral meningitis (10.8%), urinary tract infections (7.4%), sepsis (6.3%), gastroenteritis (5.9%), pneumonia (4.1%), encephalitis (3.3%), and BM (2.6%). Other diagnoses made up 16%.

### Age as a predictor of the likelihood of receiving meningitis treatment

Both age and BMS were significant predictors of receiving antibiotic treatment for BM. Older children, OR = 0.70, 95%-CI [0.53, 0.91], and children with a negative BMS were less likely to receive a treatment, OR = 0.46, 95%-CI [0.27, 078]. Adding an interaction effect between age and BMS results in a significant increase of R² (Table 3). Older children with a negative BMS were less likely to receive BM treatment than children with non-negative BMS, OR = 0.53, 95%-CI [0.29, 0.97]. A detailed analysis of the interaction effect using the Johnson-Neyman technique showed that the interaction between age and BMS was significant for children aged 1.18 years or older.

**Table 3:** Model comparison between base and interaction model

			LR-Test		Model fit	
	AIC	BIC	χ²(1)	*p*	Nakagawa et al. Marginal *R*²	McKelvey & Zavoinas *R*²
Base model	353.84	371.85			0.101	0.156
Interaction model	351.20	372.81	4.63	0.0313	0.138	0.191

LR = likelihood ratio test, AIC = Akaike information criterion, BIC = Bayesian information criterion

### Effect of bacterial meningitis and meningococcal sepsis events

To consider the effects of BM or meningococcal sepsis events within the hospital, two models were developed. In the first model, considering a 4-month period following an event, there was a significant increase in the likelihood of a LP for a child, OR = 1.19, 95%-CI [1.08, 1.30]. The second model showed no significant effect for the month when the event occurred OR = 2.06, 95%-CI [0.96, 4.45], compared to months without an event. There was no increase in the proportion of children with a negative BMS who received antibiotic treatment for BM after events of BM and meningococcal sepsis.

## Discussion

In this study, we observed a very low incidence of BM in pediatric patients. Only five cases of BM were diagnosed among 30.967 inpatients over five years. These findings are consistent with the well-documented decline of invasive BM in the era of conjugate vaccines [16], highlighting the diagnostic challenges in febrile children presenting without a clear focus of infection. The BMS demonstrated strong predictive performance in our cohort of 269 patients receiving LP, with 100% of patients with scores ≥ 3 diagnosed with BM, while the incidence was only 0.8% in BMS = 0 and 1.6% in BMS = 1. However, these children were clinically assessed as critically ill and received prompt BM-directed antibiotic therapy. This supports previous evidence that the BMS is a reliable tool for risk stratification in pediatric patients suspected of BM [17]. Nevertheless, a notable proportion of children with a negative BMS still received empirical antibiotic therapy, indicating a persistent discrepancy between score-based recommendations and real-life clinical decision-making.

A novel observation from this investigation is a significant increase in LP frequency after an event of BM or meningococcal sepsis occurred at the hospital. This effect was temporally limited to a 4-month period and did not persist. It suggests a short-lived behavioral ‘alarm’ response rather than a sustained change in diagnostic practice. These findings show that rare but dramatic infectious disease cases may temporarily influence the risk perception of clinicians, which may be also associated with an increased readiness to perform invasive diagnostic procedures, even in the absence of strong objective clinical indicators. However, these observations may be also consistent with cognitive biases such as ‘information bias’ and ‘availability bias’ [18]. Information bias describes the assumption that more information improves decision-making, even when the information is irrelevant or misleading. In this context, LP was performed in 30% of children for whom meningitis/encephalitis was not among the initial differential diagnoses upon admission, but was performed to rule out other differential diagnoses. Therefore, this finding should be interpreted with caution, as causality cannot be concluded from the study design. In addition, availability bias describes the tendency to rather choose diagnoses or events that one has recently experienced, found emotionally powerful, or particularly impressive as an explanation more often, regardless of their actual probability [19]. Importantly, these effects may consequently contribute to the higher frequency of LP following actual meningococcal infections or other BM. However, as no psychological or behavioral variables were assessed, this interpretation still remains hypothetical.

BM and bacterial sepsis exhibit a degree of seasonality [20, 21]. Despite this, our events occur throughout the year. A seasonal rise could, theoretically, exert an additional influence on the LP rate.

In contrast, an overlooked bacterial infection can have potentially serious consequences, particularly BM and meningococcal infections [22]. This knowledge may also contribute to a tendency toward more extensive diagnostic work-up and overtreatment in febrile infants, reflecting a precautionary approach in situations of clinical uncertainty [9]. Although prior events influenced the indication for LP in our study, they did not result in increased antibiotic therapy. Examination of the CSF appears to have provided sufficient certainty to rule out BM in these cases. However, the proportion of children who initially received antibiotic therapy, regardless of BMS, was relatively high across all months.

In our study, patient age was also a key factor influencing treatment decisions. Children under two years of age were significantly more likely to receive BM-targeted antibiotic therapy, even with low BMS results. The negative correlation between age and antibiotic use in BMS-negative patients suggests that clinicians may depend more confidently on structured decision tools, such as the BMS, in older children. At the same time, uncertainty remains higher in early infancy. This might also be related to the lower diagnostic accuracy of such scores, especially in young infants [23], but from our perspective, that does not fully explain the phenomenon. Other cognitive factors, such as outcome or action bias, may also influence clinicians’ ongoing concern about a missed case diagnosis [24].

The relatively short average duration of antibiotic treatment generally indicates effective implementation of de-escalation strategies in antimicrobial stewardship. Antimicrobial therapy for suspected BM is discontinued after about three days if inflammation markers, CSF tests, and microbiological findings remain negative. Here, strengthening the use of validated scores, such as the BMS, could help avoid unnecessary treatments. It may also support early discontinuation in low-risk settings. This should be included in antimicrobial stewardship programs (ASP), along with considering potentially relevant psychological factors [25]. However, treatment of confirmed BM is commonly well defined and guideline-driven, often requiring prolonged therapy depending on the pathogen and clinical course, which limits the extent to which stewardship-related conclusions can be drawn from these data. The high density of LPs relative to the low incidence of actual BM imposes a relevant burden on patients, parents, and hospital resources. Still, the procedure appears justified in 70% of cases, where there was an initial suspicion of meningitis or encephalitis. Even in the remaining 30% of cases, LP is part of the guideline-based differential diagnosis, especially in children under three months of age with a fever of unknown origin [3].

The antibiotic double coverage of BM with cephalosporins and ampicillin in children over one year of age is likely overtreatment, as infections with naturally cephalosporin-resistant *Listeria monocytogenes* or *Enterococcus* spp. are sporadic in this age group [26]. Targeted training and ASPs may help to promote guideline adherence, ensuring that double coverage with ampicillin is limited to infants and immunocompromised individuals. The high proportion of combination therapies with acyclovir for possible viral encephalitis caused by herpes simplex virus (HSV) reflects a potential overuse of antiviral treatment, given the low incidence in patients over the age of five years [27]. ASPs could also help limit acyclovir treatment to infants or patients having other neurological symptoms in addition to fever [28].

To date, only a few studies have investigated whether rare invasive infectious disease events influence clinical behavior and how long these effects persist [29, 30]. Our findings generate hypotheses for future research by suggesting a measurable, time-limited increase in invasive diagnostic procedures following cases of BM or meningococcal sepsis, highlighting the potential importance of considering subjective risk perception in everyday clinical practice. In this context, ASPs conducting routine ward audits can help emphasize objective reasoning in antimicrobial treatment. Strengthening training in “low-risk” scenarios in febrile children and clarifying the predictive value of the BMS may support more consistent decision-making. The implementation and repeated practice of structured diagnostic algorithms could further reduce inappropriate indications for LP and empirical therapy [31]. In addition, LP and antibiotic use may serve as quality indicators for diagnostic and therapeutic practice in ASPs in pediatric settings.

Future studies should include prospective assessments of psychological factors and cognitive biases, for example, by surveying clinicians’ decision pathways after sentinel events. Multicenter approaches could help capture structural and cultural differences between institutions and training settings. Moreover, it also remains to be explored whether similar short-term effects on diagnostic or therapeutic behavior occur after other rare but clinically significant conditions, such as HSV-caused encephalitis or invasive group A streptococcal infections [32]. Finally, the impact of targeted training or ASP interventions that address cognitive biases warrants systematic evaluation.

## Conclusion

In a pediatric setting with a very low incidence of BM and meningococcal sepsis, this study shows that rare invasive infectious events are associated with a short-term increase in LP use, while antibiotic treatment practices remain largely unchanged. These findings may suggest that recent high-impact cases may transiently influence diagnostic behavior, even when validated risk stratification tools are available. By integrating microbiological findings, clinical characteristics, and treatment data, this study highlights the potential relevance of cognitive factors in pediatric infectious disease management. However, these factors were not directly measured and warrant investigation in future studies. Recognizing such potential influences may help strengthen evidence-based decision-making. It may also support antimicrobial stewardship and quality improvement efforts across different healthcare settings.

### Strengths and limitations

This study has several strengths. It is based on a large cohort collected over five years and combines microbiological results, clinical diagnoses, and treatment data, allowing for a comprehensive assessment of diagnostic and therapeutic decisions. Notably, the analysis includes objective correlations with real-world cases of BM and meningococcal sepsis, offering a unique perspective on how severe infectious events may influence clinical behavior in pediatric acute care.

However, as a single-center study, the findings reflect local epidemiology, event density, and team culture, which may limit how well they are applicable in other settings. In addition, the observation period overlaps with the COVID-19 pandemic, which has affected the incidence of many infectious diseases through different containment measures. The retrospective design prevents causal conclusions, and psychological influences on decision-making were not assessed. There were no qualitative surveys were performed among clinicians. Furthermore, individual decision pathways were not systematically documented. Future studies should examine the above-mentioned cognitive factors, as well as the potential effects of parental anxiety, staffing patterns, and clinician experience. These factors may have influenced diagnostic thresholds but could not be analyzed within the current retrospective framework. Qualitative research should be used in particular to capture these factors.

## Electronic Supplementary Material

Below is the link to the electronic supplementary material.

Supplementary Material 1

## Data Availability

The datasets used and/or analyzed during the current study are available from the corresponding author on reasonable request.

## Abbreviations

ASP: Antimicrobial stewardship programs
BM: Bacterial meningitis
BMS: Bacterial Meningitis Score
CI: Confidence interval
CSF: Cerebrospinal fluid
COVID-19: Coronavirus disease 2019
HSV: Herpes simplex virus
LP: Lumbar puncture
OR: Odds ratio
SD: Standard deviation
