# Supplementary material for: Lumbar puncture use and antibiotic treatment in newly admitted febrile children following index cases of invasive infections
Source: BMC Infect Dis. 2026 May 20;26:987. doi: 10.1186/s12879-026-13486-1 (PMC13196126; doi:10.1186/s12879-026-13486-1)
Supplement: Supplementary file 1 — Supplementary Material 1 [file 12879_2026_13486_MOESM1_ESM.html]

Lumbar Puncture Use and Antibiotic Treatment in Newly Admitted Febrile Children Following Index Cases of Invasive Infections


## Table of contents

- 1. Setup
- 2. Data import
- 3. Data preparation
- 4. Demographics
- 5. Descriptives
  - 5.1 Lumbar puncture
  - 5.2 Meningitis events
  - 5.3 Meningitis therapy
  - 5.4 BMS
  - 5.5 Therapy duration
- 6. Tests
  - 6.1 Age differences
  - 6.2 Age & BMS as predictor of meningitis treatment
  - 6.3 Lumbar punction after event
- 7. Plots for publication

# Lumbar Puncture Use and Antibiotic Treatment in Newly Admitted Febrile Children Following Index Cases of Invasive Infections

Digital supplemental material and full R code

Authors

Maximilian David Mauritz, MD, MSc

Matthias Sehlbrede, MSc

Julia Wager, PhD

Malik Aydin, MD, PhD

Published

April 15, 2026

Content note

This supplemental material contains all analysis conducted in R. Some of the descriptive statistics seen in Tables 1 and 2 were conducted in SPSS and are not shown here. All statistical tests were conducted in R and are shown here.

# 1. Setup

- R Packages
- Session Info
- Code informations

Code

```
suppressPackageStartupMessages(if(!require(pacman)) {install.packages("pacman")})
pacman::p_load(rio, dplyr, tidyr, gt, emmeans,
               ggplot2, sjPlot, effectsize,
               lme4, lmerTest, glmmTMB, interactions, 
               rstatix, ggtext, performance)
```

Code

```
sessionInfo()
```

```
R version 4.4.2 (2024-10-31 ucrt)
Platform: x86_64-w64-mingw32/x64
Running under: Windows 11 x64 (build 26100)

Matrix products: default


locale:
[1] LC_COLLATE=German_Germany.utf8  LC_CTYPE=German_Germany.utf8   
[3] LC_MONETARY=German_Germany.utf8 LC_NUMERIC=C                   
[5] LC_TIME=German_Germany.utf8    

time zone: Europe/Berlin
tzcode source: internal

attached base packages:
[1] stats     graphics  grDevices utils     datasets  methods   base     

other attached packages:
 [1] performance_0.15.2 ggtext_0.1.2       rstatix_0.7.3      interactions_1.2.0
 [5] glmmTMB_1.1.13     lmerTest_3.1-3     lme4_1.1-37        Matrix_1.7-4      
 [9] effectsize_1.0.1   sjPlot_2.9.0       ggplot2_4.0.1      emmeans_2.0.0     
[13] gt_1.1.0           tidyr_1.3.1        dplyr_1.1.4        rio_1.2.4         
[17] pacman_0.5.1      

loaded via a namespace (and not attached):
 [1] tidyselect_1.2.1    farver_2.1.2        S7_0.2.1           
 [4] fastmap_1.2.0       bayestestR_0.17.0   jtools_2.3.0       
 [7] digest_0.6.38       estimability_1.5.1  lifecycle_1.0.4    
[10] magrittr_2.0.4      compiler_4.4.2      rlang_1.1.6        
[13] tools_4.4.2         yaml_2.3.10         knitr_1.50         
[16] htmlwidgets_1.6.4   xml2_1.4.1          RColorBrewer_1.1-3 
[19] abind_1.4-8         withr_3.0.2         purrr_1.2.0        
[22] numDeriv_2016.8-1.1 grid_4.4.2          datawizard_1.3.0   
[25] xtable_1.8-4        future_1.68.0       globals_0.18.0     
[28] scales_1.4.0        MASS_7.3-65         insight_1.4.2      
[31] cli_3.6.5           mvtnorm_1.3-3       rmarkdown_2.30     
[34] reformulas_0.4.2    generics_0.1.4      rstudioapi_0.17.1  
[37] parameters_0.28.2   minqa_1.2.8         pander_0.6.6       
[40] splines_4.4.2       parallel_4.4.2      vctrs_0.6.5        
[43] boot_1.3-32         sandwich_3.1-1      carData_3.0-5      
[46] jsonlite_2.0.0      car_3.1-3           Formula_1.2-5      
[49] listenv_0.10.0      glue_1.8.0          parallelly_1.45.1  
[52] nloptr_2.2.1        codetools_0.2-20    gtable_0.3.6       
[55] broom.mixed_0.2.9.6 tibble_3.3.0        pillar_1.11.1      
[58] furrr_0.3.1         htmltools_0.5.8.1   R6_2.6.1           
[61] TMB_1.9.18          Rdpack_2.6.4        evaluate_1.0.5     
[64] lattice_0.22-7      rbibutils_2.4       backports_1.5.0    
[67] gridtext_0.1.5      broom_1.0.10        Rcpp_1.1.0         
[70] coda_0.19-4.1       nlme_3.1-168        mgcv_1.9-4         
[73] xfun_0.54           fs_1.6.6            zoo_1.8-14         
[76] forcats_1.0.1       pkgconfig_2.0.3
```

M. Sehlbrede was responsible for the analysis conducted in R. All notes were translated into englisch, some variable names and labels remained in germany, because the initial script was in german. We tried different kind of figures for publication (chapter 7) but this script only includes the one, we finally used. The original script was re-formatted to increase readibility and structure when used as supplemental material.

# 2. Data import

Code

```
df_raw <- import("LP export.xlsx")
patientenzahlen <- import("Patientenzahlen_aufbereitet.csv", encoding = "Latin-1")
df <- df_raw
```

Two data frames are importes: the first data set contains cases (N = 269), all with lumbar puncture alongside with informations about BMS, therapy duration and meningities therapy. The second data set contains aggregated total number of cases aggregated on clinical station level per month for the years 2019 to 2024.

# 3. Data preparation

Code

```
# renaming (german names still in use)
names(df) <- c("Gender", "Fallnr", "Aufnahme_Datum", "Entlassung_Datum",
               "Aufnahmemonat_Jahr",
               "Art", "Verweildauer", "Fachabteilung", "station", "Date", "Lei_Zeit",
               "OP_Code", "Init_MeningV", "BMS", "Meningthera", "Therapiedauer_Tage", 
               "Thera_Art", "Entlass_Diag", "Diagnosegruppe", "Age", "Erreger_Liquor",
               "Erreger_BK")
# factorisation
df <- df |> 
  mutate(
    Gender = factor(Gender,
                        levels = c("M", "W"),
                        labels = c("male", "female")),#
    # Initialer Meningitsverdacht
    Init_MeningV = factor(Init_MeningV,
                          levels = c(0, 1),
                          labels = c("no", "yes")))
  
# create new time variable seperate month from year base on date
df <- df |>
  mutate(
    month = gsub("[[:digit:]]{4}-|-.*", "", Aufnahme_Datum),
    year = gsub("-.*", "", Date))

# coding blood culture: was a positive effect found?
# NA values represents "no", for those we impute a zero. positive cases
# remained coded with 1
df$pathogen <- ifelse(is.na(df$Erreger_BK), "0", 1)
```

All of *N* = 269 cases got lumbar puncture so for later use, to avoid a contant dependend variable we had to add all children without lumbar puncture. The primary data was not aggregated, each case had its own row. First we add a dummy variable for lumbar puncture (1 = yes, 0 = no). Then we aggregated the primary data for each month and year and then we add the total patient data set, which was already aggregated on those levels.

Code

```
df_lumbal <- df |> 
  select(station, month, year, pathogen) |> 
  # create new dummy variable, all cases coded 1 for lumbar puncture = yes
  mutate(LP = 1) |> 
  group_by(station, month, year) |> 
  mutate(pathogen = as.numeric(pathogen)) |> 
  summarise(pathogen = sum(pathogen),
            LP = sum(LP),
            .groups = "drop")


df_pat <- patientenzahlen |> 
  rename(year = Jahr, month = Monat) |> 
  pivot_longer(cols = -c(year, month),
               names_to = "station",
               values_to = "Pat_Total") |> 
  mutate(station = case_when(station == "Station_1A" ~ "ST-1A",
                             station == "Station_3A" ~ "ST-3A",
                             station == "Station_3D" ~ "ST-3D")) |> 
  mutate(month = case_when(month == "Januar" ~ "01",
                           month == "Februar" ~ "02",
                           month == "März" ~ "03",
                           month == "April" ~ "04",
                           month == "Mai" ~ "05",
                           month == "Juni" ~ "06",
                           month == "Juli" ~ "07",
                           month == "August" ~ "08",
                           month == "September" ~ "09",
                           month == "Oktober" ~ "10",
                           month == "November" ~ "11",
                           month == "Dezember" ~ "12",))

# merge both data sets
df_allpat <- merge(df_lumbal, df_pat, by = c("station", "year", "month"),
                   all = TRUE)
# impute zero if no meningitis event
df_allpat <- df_allpat |>
  mutate(across(c(pathogen, LP), function(x) {
    ifelse(is.na(x), 0, x)
  }))
```

Additional a second data set was created for the analysis shown in Section 6.2: we took the number of cases from the full patient data set and create a wide-foramt data set. Each case got its own dummy coded lumbar puncture value alongside with dummy coded meningitis event. We added two differend time coded variables: first one (time) codes time scale\_y\_) for each month startet with 0 (januar) to 11 (december) the other codes a timeframe over 4 month (TaEvent) coded 0 to 4.

Code

```
.info_cases <- df_allpat |>
  mutate(ohne_LP = Pat_Total-LP) |> 
  select(year, month, LP, ohne_LP) |> 
  group_by(year, month) |> 
  summarise(LP = sum(LP, na.rm = TRUE),
            ohne_LP = sum(ohne_LP, na.rm = TRUE),
            .groups = "drop") |> 
  arrange(year, month) 

.df_casetotal <- lapply(unique(.info_cases$year), function(y) {
  
  df <- .info_cases[.info_cases$year == y,]

  lapply(df$month, function(x) {

    df2 <- data.frame(
      LP = c(
        rep(1, df[df$month == x, "LP"]),
        rep(0, df[df$month == x, "ohne_LP"])
      ),
      month = rep(x, sum(df[df$month == x, "LP"],
                         df[df$month == x, "ohne_LP"]))
    )
    df2 |> 
      mutate(year = rep(y, nrow(df2)))
  })
  
})

df_casetotal <- do.call("bind_rows", .df_casetotal)
df_casetotal$Id <- paste("id_", 1:nrow(df_casetotal), sep = "")

df_casetotal <- merge(
  df_casetotal,
  df_allpat |> select(month, year, pathogen) |> filter(pathogen == 1),
  by = c("month", "year"),
  all = TRUE
)

df_casetotal <- df_casetotal |> 
  mutate(pathogen = ifelse(is.na(pathogen), 0, pathogen))
# addint time 0 to 11 for each month
df_casetotal$time <- as.numeric(df_casetotal$month)-1

.df_mod <- df_allpat |> 
  group_by(month, year) |> 
  summarise(LP = sum(LP),
            pathogen = sum(pathogen, na.rm = TRUE),
            Pat_Total = sum(Pat_Total, na.rm = TRUE),
            .groups = "drop") |> 
  mutate(p_LP = LP / Pat_Total)

.df_mod <- .df_mod |> 
  arrange(year, month) |> 
  mutate(time = as.numeric(month)-1) |> 
  mutate(TaE = case_when(pathogen == 0 ~ 0,
                         pathogen == 1 ~ 1)) |> 
  mutate(TaEvent = TaE)

for(i in which(.df_mod$TaEvent == 1)) {
  
  if(i + 3 <=length(.df_mod$TaEvent)) {
    
    .df_mod$TaEvent[i:(i+3)] <- 1:4
    
  }
  
}

df_casetotal <- merge(df_casetotal,
                      .df_mod |> dplyr::select(month, year, TaEvent),
                      by = c("month", "year"))
```

Seperate data set for BMS analysis based on main lumbar puncture data set. BMS: bactieral meningitis score (0 = no meningitis)

Code

```
# create BMS data set based only on the n = 269 case data set
# (= only cases with lumbar puncture)
df_BMS <- df |> 
  dplyr::select(Fallnr, month, year, Age, 
                pathogen, BMS, Meningthera) |> 
  mutate(Meningthera = ifelse(is.na(Meningthera), 0, Meningthera)) |> 
  mutate(time = as.numeric(month)-1)
# create BMS dummy variable
# BMS = 0 vs. BMS > 0
df_BMS <- df_BMS |> 
  mutate(BMS_jn = case_when(BMS == 0 ~ 1,
                            BMS > 0 ~ 0))
# create categorical age variable
df_BMS <- df_BMS |> 
  mutate(Alter_cat = case_when(Age  < 2 ~ "0-2",
                               Age  >= 2 & Age  < 6 ~ "2-6",
                               Age  > 6 ~ "6-18"))
# adding therapy duration
df_BMS <- merge(
  df_BMS,
  df |> dplyr::select(Fallnr, Therapiedauer_Tage),
  by = "Fallnr", all.x = TRUE)
```

# 4. Demographics

Code

```
rbind(
  df |> 
    summarise(
      n = length(na.omit(Age)),
      M = mean(Age, na.rm = TRUE),
      SD = sd(Age, na.rm = TRUE),
      Min = min(Age, na.rm = TRUE),
      Max = max(Age, na.rm = TRUE),
      under1 = sum(Age < 1, na.rm = TRUE),
      punder1 = sum(Age < 1, na.rm = TRUE) / length(na.omit(Age)),
    ) |> 
    mutate(Gender = "Total",
           p = "100.00%"),
  df |> 
    group_by(Gender) |> 
    summarise(
      n = length(na.omit(Age)),
      M = mean(Age, na.rm = TRUE),
      SD = sd(Age, na.rm = TRUE),
      Min = min(Age, na.rm = TRUE),
      Max = max(Age, na.rm = TRUE),
      under1 = sum(Age < 1, na.rm = TRUE),
      punder1 = sum(Age < 1, na.rm = TRUE) / length(na.omit(Age)),
    ) |> 
    mutate(p = paste(round(n / sum(n)*100, 2), "%", sep = ""))
  ) |> 
  mutate(np = paste(n, " (", p, ")", sep = "")) |> 
  dplyr::select(Gender, np, M : Max, under1, punder1) |> 
  gt() |> 
  cols_label(
    np = "n Cases (%)",
    under1 = "n",
    punder1 = "%"
  ) |> 
  fmt_number(columns = c(M, SD, Min, Max), decimals = 2) |> 
  tab_spanner(columns = c(M, SD, Min, Max), "Age statistics") |> 
  tab_spanner(columns = c(under1, punder1), "Cases < 1 year") |> 
  fmt_percent(columns = punder1, decimals = 2)
```

Table 1: Sample demographics

| Gender | n Cases (%) | Age statistics | | | | Cases < 1 year | |
| --- | --- | --- | --- | --- | --- | --- | --- |
|  |  | M | SD | Min | Max | n | % |
| Total | 269 (100.00%) | 2.90 | 4.25 | 0.01 | 17.73 | 142 | 52.79% |
| male | 159 (59.11%) | 2.76 | 4.01 | 0.04 | 17.73 | 85 | 53.46% |
| female | 110 (40.89%) | 3.11 | 4.57 | 0.01 | 17.48 | 57 | 51.82% |

# 5. Descriptives

Not in this code

Following statistics were calculated in SPSS and are not shown here:

- Suspected bacterial meningitis and antibiotic therapies (Paper Table 1)
- Pathogens detected in cerebrospinal fluid and blood cultures (Paper Table 2)

## 5.1 Lumbar puncture

- prevalence
- over time

Code

```
# prevalence total
praev.total <- df_casetotal |> 
  summarise(N = length(LP),
            LP = sum(LP == 1)) |> 
  mutate(praev = LP / N,
         year = "Overall")
# prevalence for each year
praev.year <- df_casetotal |> 
  group_by(year) |> 
  summarise(N = length(LP),
            LP = sum(LP == 1)) |> 
  mutate(praev = LP / N)

rbind(praev.total, praev.year) |> 
  dplyr::select(year, everything()) |> 
  gt() |> 
  fmt_percent(columns = "praev", decimals = 3)
```

Figure 1: Prevalence of lumbar puncture overall and for each year

| year | N | LP | praev |
| --- | --- | --- | --- |
| Overall | 30967 | 269 | 0.869% |
| 2019 | 5697 | 51 | 0.895% |
| 2020 | 4198 | 24 | 0.572% |
| 2021 | 4433 | 27 | 0.609% |
| 2022 | 5415 | 40 | 0.739% |
| 2023 | 5536 | 72 | 1.301% |
| 2024 | 5688 | 55 | 0.967% |

Code

```
df_allpat |> 
  group_by(month, year) |> 
  summarise(LP = sum(LP),
            pathogen = sum(pathogen, na.rm = TRUE),
            Pat_Total = sum(Pat_Total, na.rm = TRUE),
            .groups = "drop") |> 
  mutate(p_LP = LP / Pat_Total) |> 
  arrange(year, month) |> 
  ggplot(aes(x = month, y = p_LP)) +
  facet_wrap(~ year, ncol = 2) +
  geom_col() +
  geom_point(aes(y = 0, color = factor(pathogen)),
             shape = 15, size = 3) +
  geom_text(aes(label = paste(Pat_Total, "\n(", LP, ")", sep = "")),
            vjust = -0.2) +
  scale_y_continuous(labels = scales::percent_format(accuracy = 1),
                     limits = c(0, 0.05)) +
  scale_color_manual(values = c("steelblue", "orange"),
                     labels = c("No event", "Meningitis event")) +
  theme_classic() +
  theme(legend.position = "bottom") +
  labs(x = "Month",
       y = "Frequency [%]",
       color = "",
       caption = "Labels: Total number of cases (cases with lumbar puncture)")
```

Figure 2: Frequency of lumbar puncture

## 5.2 Meningitis events

- frequency
- by age

Code

```
df |> 
  group_by(year) |> 
  count(pathogen) |> 
  filter(pathogen == 1) |> 
  dplyr::select(-pathogen) |> 
  ungroup() |> 
  gt() |> 
  cols_label(n = "Frequency of meningitis events",
             year = "Year")
```

Table 2: Frequency of meningitis events grouped by year

| Year | Frequency of meningitis events |
| --- | --- |
| 2019 | 1 |
| 2022 | 1 |
| 2023 | 3 |
| 2024 | 1 |

Code

```
df |> 
  mutate(Alter_cat = case_when(Age  < 2 ~ "0-2",
                               Age  >= 2 & Age  < 6 ~ "2-6",
                               Age  > 6 ~ "6-18")) |> 
  summarise(S = sum(pathogen == 1), .by = "Alter_cat") |> 
  gt() |> 
  cols_label(S = "Frequency of meningitis events",
             Alter_cat = "Age category")
```

Table 3: Frequency of meningitis events grouped per age

| Age category | Frequency of meningitis events |
| --- | --- |
| 0-2 | 6 |
| 2-6 | 0 |
| 6-18 | 0 |

## 5.3 Meningitis therapy

- over time
- by age groups
- by BMS and age groups

Code

```
.plot_df <- df |> 
  select(month, year, pathogen, Meningthera) |> 
  mutate(Meningthera = ifelse(is.na(Meningthera), 0, Meningthera)) |> 
  group_by(month, year, pathogen) |> 
  count(Meningthera) |> 
  group_by(month, year) |>
  mutate(N = sum(n))

ggplot(data = .plot_df |> dplyr::select(-Meningthera, -n) |> unique(),
       mapping = aes(x = month, y = N, fill = "A")) +
  facet_wrap(~ year) +
  geom_col() +
  geom_col(
    data = .plot_df |> filter(Meningthera == 1),
    mapping = aes(x = month, y = n, fill = "B", width = .5)
  ) +
  geom_point(aes(y = 0, color = factor(pathogen)),
             shape = 15, size = 3) +
  scale_color_manual(values = c("steelblue", "orange"),
                     labels = c("No event", "Meningitis event")) +
  scale_fill_manual(values = c("#1E1E1E", "#ABABAB"),
                    labels = c("All children", "Children with meningitis therapy")) +
  labs(x = "Month",
       y = "Frequency [n]",
       fill = "",
       color = "") +
  theme_classic() +
  theme(legend.position = "bottom")
```

Figure 3: Frequency of children with lubar puncuture and meningitis therapy

Code

```
df_BMS |> 
  group_by(Alter_cat) |> 
  count(Meningthera) |> 
  mutate(N = sum(n)) |> 
  mutate(n = paste(
    n,
    " (",
    format(round(n / sum(n)*100, 1), nsmall = 1),
    "%)",
    sep = ""
  )) |> 
  pivot_wider(names_from = Meningthera, values_from = n) |> 
  ungroup() |> 
  gt(rowname_col = "Alter_cat") |> 
  tab_stubhead(label = "Age category") |> 
  cols_label(`0` = "no",
             `1` = "yes") |> 
  cols_align(align = c("left"),
             columns = c("Alter_cat")) |> 
  cols_width(
    Alter_cat ~ px(150),
    `0` ~ px(100),
    `1` ~ px(100)
  ) |> 
  tab_spanner(columns = c(`0`, `1`),
              label = "Meningits therapy")
```

Table 4: Frequency of meningitis therapy grouped by age

| Age category | N | Meningits therapy | |
| --- | --- | --- | --- |
|  |  | no | yes |
| 0-2 | 177 | 71 (40.1%) | 106 (59.9%) |
| 2-6 | 44 | 26 (59.1%) | 18 (40.9%) |
| 6-18 | 50 | 31 (62.0%) | 19 (38.0%) |

Code

```
df_BMS |> 
  group_by(Alter_cat) |> 
  count(Meningthera, BMS_jn) |> 
  mutate(n = paste(
    n,
    " (",
    format(round(n / sum(n)*100, 1), nsmall = 1),
    "%)",
    sep = ""
  )) |> 
  pivot_wider(names_from = Meningthera, values_from = n) |> 
  # BMS = 0 ist so kodiert, dass ein BMS von 0 = ja (kodiert  als 1)
  # bedeutet!
  mutate(BMS_jn = factor(BMS_jn,
                         levels = c(0, 1),
                         labels = c("no", "yes"))) |> 
  ungroup() |> 
  gt(rowname_col = "Alter_cat") |> 
  tab_stubhead(label = "Age category") |> 
  cols_label(`0` = "no",
             `1` = "yes",
             BMS_jn = "BMS = 0") |> 
  cols_align(align = c("left"),
             columns = c("Alter_cat")) |> 
  cols_width(
    Alter_cat ~ px(150),
    `0` ~ px(100),
    `1` ~ px(100)
  ) |> 
  tab_spanner(columns = c(`0`, `1`),
              label = "Meningitis therapy")
```

Table 5: Frequency of meningitis therapy grouped for age and BMS

| Age category | BMS = 0 | Meningitis therapy | |
| --- | --- | --- | --- |
|  |  | no | yes |
| 0-2 | no | 33 (18.6%) | 61 (34.5%) |
| 0-2 | yes | 38 (21.5%) | 45 (25.4%) |
| 2-6 | no | 16 (36.4%) | 15 (34.1%) |
| 2-6 | yes | 10 (22.7%) | 3 ( 6.8%) |
| 6-18 | no | 8 (16.0%) | 15 (30.0%) |
| 6-18 | yes | 23 (46.0%) | 4 ( 8.0%) |

## 5.4 BMS

- frequency
- over time

Code

```
df |> 
  select(month, year, pathogen, BMS) |> 
  count(BMS) |> 
  mutate(p = n / sum(n)) |> 
  gt() |> 
  fmt_percent(columns = p, decimals = 2)
```

Table 6: Frequency of BMS

| BMS | n | p |
| --- | --- | --- |
| 0 | 121 | 44.98% |
| 1 | 123 | 45.72% |
| 2 | 22 | 8.18% |
| 3 | 2 | 0.74% |
| 4 | 1 | 0.37% |

Code

```
.plot_df <- df |> 
  select(month, year, pathogen, BMS) |> 
  group_by(month, year, pathogen) |> 
  count(BMS) |> 
  group_by(month, year) |>
  mutate(N = sum(n))

ggplot(data = .plot_df |> dplyr::select(-BMS, -n) |> unique(),
       mapping = aes(x = month, y = N, fill = "A")) +
  facet_wrap(~ year) +
  geom_col() +
  geom_col(
    data = .plot_df |> filter(BMS == 0),
    mapping = aes(x = month, y = n, fill = "B", width = .5)
  ) +
  geom_point(aes(y = 0, color = factor(pathogen)),
             shape = 15, size = 3) +
  scale_color_manual(values = c("steelblue", "orange"),
                     labels = c("No event", "Meningitis event")) +
  scale_fill_manual(values = c("#1E1E1E", "#ABABAB"),
                    labels = c("All children", "Children with BMS = 0")) +
  labs(x = "Month",
       y = "Frequency [n]",
       fill = "",
       color = "") +
  theme_classic() +
  theme(legend.position = "bottom")
```

Figure 4: Frequency of children with lubar puncture and BMS = 0

## 5.5 Therapy duration

- global
- by age group
- by BMS

Code

```
df_BMS |> 
  filter(Meningthera == 1) |> 
  summarise(N = length(na.omit(Therapiedauer_Tage)),
            M = mean(Therapiedauer_Tage, na.rm = TRUE),
            SD = sd(Therapiedauer_Tage, na.rm = TRUE),
            Min = min(Therapiedauer_Tage, na.rm = TRUE),
            Q25 = quantile(Therapiedauer_Tage, .25, na.rm = TRUE),
            Median = median(Therapiedauer_Tage, na.rm = TRUE),
            Q75 = quantile(Therapiedauer_Tage, .75, na.rm = TRUE),
            Max = max(Therapiedauer_Tage, na.rm = TRUE)) |> 
  gt() |> 
  fmt_number(columns = c("M", "SD"), decimals = 2)
```

Table 7: Therapy duration for children with meningitis therapy

| N | M | SD | Min | Q25 | Median | Q75 | Max |
| --- | --- | --- | --- | --- | --- | --- | --- |
| 142 | 3.82 | 2.79 | 1 | 2 | 3 | 5 | 21 |

Code

```
df_BMS |> 
  filter(Meningthera == 1) |> 
  group_by("Age group" = Alter_cat) |> 
  summarise(N = length(na.omit(Therapiedauer_Tage)),
            M = mean(Therapiedauer_Tage, na.rm = TRUE),
            SD = sd(Therapiedauer_Tage, na.rm = TRUE),
            Min = min(Therapiedauer_Tage, na.rm = TRUE),
            Q25 = quantile(Therapiedauer_Tage, .25, na.rm = TRUE),
            Median = median(Therapiedauer_Tage, na.rm = TRUE),
            Q75 = quantile(Therapiedauer_Tage, .75, na.rm = TRUE),
            Max = max(Therapiedauer_Tage, na.rm = TRUE)) |> 
  gt() |> 
  fmt_number(columns = c("M", "SD"), decimals = 2)
```

Table 8: Therapy duration for children with meningits therapy grouped by age

| Age group | N | M | SD | Min | Q25 | Median | Q75 | Max |
| --- | --- | --- | --- | --- | --- | --- | --- | --- |
| 0-2 | 106 | 3.78 | 2.79 | 1 | 2 | 3 | 5 | 21 |
| 2-6 | 17 | 3.76 | 1.92 | 1 | 2 | 3 | 5 | 7 |
| 6-18 | 19 | 4.11 | 3.49 | 1 | 2 | 3 | 6 | 14 |

Info: some on therapy duration was missing for the analysis, so the frequency did not add up to N = 269!

Code

```
df_BMS |> 
  filter(Meningthera == 1) |> 
  group_by(BMS) |> 
  summarise(N = length(na.omit(Therapiedauer_Tage)),
            M = mean(Therapiedauer_Tage, na.rm = TRUE),
            SD = sd(Therapiedauer_Tage, na.rm = TRUE),
            Min = min(Therapiedauer_Tage, na.rm = TRUE),
            Q25 = quantile(Therapiedauer_Tage, .25, na.rm = TRUE),
            Median = median(Therapiedauer_Tage, na.rm = TRUE),
            Q75 = quantile(Therapiedauer_Tage, .75, na.rm = TRUE),
            Max = max(Therapiedauer_Tage, na.rm = TRUE)) |> 
  gt() |> 
  fmt_number(columns = c("M", "SD"), decimals = 2)
```

Table 9: Therapy duration for children with meningitis therapy grouped by BMS value

| BMS | N | M | SD | Min | Q25 | Median | Q75 | Max |
| --- | --- | --- | --- | --- | --- | --- | --- | --- |
| 0 | 52 | 3.96 | 2.63 | 1 | 2.0 | 3 | 5.0 | 14 |
| 1 | 72 | 3.11 | 1.67 | 1 | 2.0 | 3 | 4.0 | 9 |
| 2 | 15 | 5.13 | 3.38 | 1 | 3.0 | 5 | 7.0 | 14 |
| 3 | 2 | 14.00 | 9.90 | 7 | 10.5 | 14 | 17.5 | 21 |
| 4 | 1 | 8.00 | NA | 8 | 8.0 | 8 | 8.0 | 8 |

# 6. Tests

## 6.1 Age differences

No significant main effect (descriptive table see Table 8), so we dissmissed post hoc tests. Note: effect size eta squared is only an approximation due to heteroscedasticity.

Code

```
fit <- oneway.test(Therapiedauer_Tage ~ Alter_cat, 
                   data = df_BMS |> filter(Meningthera == 1))

data.frame(

  df1 = fit$parameter[[1]],
  df2 = fit$parameter[[2]],
  F_value = fit$statistic,
  p_value = fit$p.value,
  eta2 = effectsize::eta_squared(fit, var.equal = FALSE)$Eta2
  
) |> 
  gt() |> 
  cols_label(
    F_value = "F",
    p_value = "p"
  ) |> 
  fmt_number(columns = c(1:3), decimals = 2) |> 
  fmt_number(columns = c(4, 5), decimals = 3)
```

Table 10: Results of Welch-ANOVA for the test in difference between therapy duration due to age groups for children with meningitis therapy

| df1 | df2 | F | p | eta2 |
| --- | --- | --- | --- | --- |
| 2.00 | 31.41 | 0.08 | 0.927 | 0.005 |

## 6.2 Age & BMS as predictor of meningitis treatment

- Model summary
- Model comparison
- R² model fit
- Simple slopes
- Alternative model approach

- BMS\_jn was coded: “yes” (1) for BMS = 0, “no” (0) for BMS > 0
- Meningitis therapy was coded 1 = yes, 0 = no therapy

Code

```
fit_mod_base <- glmer(Meningthera ~ time + Age + BMS_jn + (1 | year), 
                      data = df_BMS,
                    family = binomial())
fit_mod_int <-  glmer(Meningthera ~ time + Age * BMS_jn + (1 | year), 
                      data = df_BMS,
                      family = binomial())

sjPlot::tab_model(fit_mod_base, fit_mod_int,
                  show.se = TRUE,
                  dv.labels = c("Model without interaction", 
                                "Mode with interaction"))
```

|  | Model without interaction | | | | Mode with interaction | | | |
| --- | --- | --- | --- | --- | --- | --- | --- | --- |
| Predictors | Odds Ratios | std. Error | CI | p | Odds Ratios | std. Error | CI | p |
| (Intercept) | 1.22 | 0.43 | 0.61 – 2.45 | 0.568 | 1.00 | 0.36 | 0.49 – 2.03 | 0.998 |
| time | 1.09 | 0.04 | 1.01 – 1.18 | **0.025** | 1.09 | 0.04 | 1.01 – 1.18 | **0.025** |
| Age | 0.92 | 0.03 | 0.86 – 0.98 | **0.009** | 0.98 | 0.05 | 0.90 – 1.08 | 0.719 |
| BMS jn | 0.46 | 0.12 | 0.27 – 0.78 | **0.004** | 0.68 | 0.22 | 0.36 – 1.28 | 0.230 |
| Age × BMS jn |  |  |  |  | 0.86 | 0.06 | 0.75 – 0.99 | **0.040** |
| Random Effects | | | | | | | | |
| σ2 | 3.29 | | | | 3.29 | | | |
| τ00 | 0.25 year | | | | 0.22 year | | | |
| ICC | 0.07 | | | | 0.06 | | | |
| N | 6 year | | | | 6 year | | | |
| Observations | 271 | | | | 271 | | | |
| Marginal R2 / Conditional R2 | 0.101 / 0.164 | | | | 0.138 / 0.192 | | | |

Likelihood ratio test (LR-Test) to compare baseline model with interaction model.

Code

```
# LR Test
anova(fit_mod_base, fit_mod_int)
```

```
Data: df_BMS
Models:
fit_mod_base: Meningthera ~ time + Age + BMS_jn + (1 | year)
fit_mod_int: Meningthera ~ time + Age * BMS_jn + (1 | year)
             npar    AIC    BIC  logLik -2*log(L)  Chisq Df Pr(>Chisq)  
fit_mod_base    5 353.84 371.85 -171.92    343.84                       
fit_mod_int     6 351.20 372.81 -169.60    339.20 4.6362  1     0.0313 *
---
Signif. codes:  0 '***' 0.001 '**' 0.01 '*' 0.05 '.' 0.1 ' ' 1
```

Code

```
performance::r2_nakagawa(fit_mod_base)
```

```
# R2 for Mixed Models

  Conditional R2: 0.164
     Marginal R2: 0.101
```

Code

```
performance::r2_nakagawa(fit_mod_int)
```

```
# R2 for Mixed Models

  Conditional R2: 0.192
     Marginal R2: 0.138
```

McKelvey & Zavoinas R² added after peer review:

Code

```
performance::r2_mckelvey(fit_mod_base)
```

```
McKelvey's R2 
    0.1568064
```

Code

```
performance::r2_mckelvey(fit_mod_int)
```

```
McKelvey's R2 
    0.1905544
```

- For age between 0 to 1.18 years, there was no significant association between BMS and meningitis therapy
- For age above 1.18, the change of getting a meningits therapy decreases when BMS was zero

Code

```
interactions::sim_slopes(fit_mod_int, pred = "Age", modx = "BMS_jn",
                         johnson_neyman = FALSE)
```

```
SIMPLE SLOPES ANALYSIS

Slope of Age when BMS_jn = 0.00 (0): 

   Est.   S.E.   z val.      p
------- ------ -------- ------
  -0.02   0.05    -0.36   0.72

Slope of Age when BMS_jn = 1.00 (1): 

   Est.   S.E.   z val.      p
------- ------ -------- ------
  -0.16   0.06    -2.96   0.00
```

Code

```
interactions::sim_slopes(fit_mod_int, pred = "BMS_jn", modx = "Age", 
                         modx.values = c(0, .5, 1, 1.5, 2, 6),
                         johnson_neyman = TRUE, jnplot = TRUE)
```

```
JOHNSON-NEYMAN INTERVAL

When Age is OUTSIDE the interval [-123.90, 1.18], the slope of BMS_jn is p
< .05.

Note: The range of observed values of Age is [0.01, 17.73]
```

```
SIMPLE SLOPES ANALYSIS

Slope of BMS_jn when Age = 0.00: 

   Est.   S.E.   z val.      p
------- ------ -------- ------
  -0.39   0.32    -1.20   0.23

Slope of BMS_jn when Age = 0.50: 

   Est.   S.E.   z val.      p
------- ------ -------- ------
  -0.46   0.30    -1.51   0.13

Slope of BMS_jn when Age = 1.00: 

   Est.   S.E.   z val.      p
------- ------ -------- ------
  -0.54   0.29    -1.84   0.07

Slope of BMS_jn when Age = 1.50: 

   Est.   S.E.   z val.      p
------- ------ -------- ------
  -0.61   0.28    -2.18   0.03

Slope of BMS_jn when Age = 2.00: 

   Est.   S.E.   z val.      p
------- ------ -------- ------
  -0.68   0.27    -2.50   0.01

Slope of BMS_jn when Age = 6.00: 

   Est.   S.E.   z val.      p
------- ------ -------- ------
  -1.28   0.38    -3.39   0.00
```

After review: model without random structure.

Code

```
.df_BMS_new <- df_BMS |> 
  mutate(year = case_when(year == 2019 ~ 1,
                          year == 2020 ~ 2,
                          year == 2021 ~ 3,
                          year == 2022 ~ 4,
                          year == 2023 ~ 5,
                          year == 2024 ~ 6)) |> 
  mutate(time_new = as.numeric(month)*year-1)

fit_alternative_base <- glm(Meningthera ~ time_new + Age + BMS_jn, 
                            data = .df_BMS_new, family = binomial)
fit_alternative_int <- glm(Meningthera ~ time_new + Age * BMS_jn, 
                           data = .df_BMS_new, family = binomial)
tab_model(fit_alternative_base, fit_alternative_int)
```

|  |  |  |  |  |  |  |
| --- | --- | --- | --- | --- | --- | --- |
|  | Meningthera | | | Meningthera | | |
| Predictors | Odds Ratios | CI | p | Odds Ratios | CI | p |
| (Intercept) | 0.94 | 0.56 – 1.57 | 0.806 | 0.76 | 0.44 – 1.31 | 0.326 |
| time new | 1.03 | 1.02 – 1.05 | **<0.001** | 1.03 | 1.02 – 1.05 | **<0.001** |
| Age | 0.92 | 0.86 – 0.98 | **0.009** | 0.99 | 0.91 – 1.09 | 0.851 |
| BMS jn | 0.50 | 0.30 – 0.83 | **0.008** | 0.76 | 0.41 – 1.41 | 0.377 |
| Age × BMS jn |  |  |  | 0.85 | 0.73 – 0.97 | **0.026** |
| Observations | 271 | | | 271 | | |
| R2 Tjur | 0.125 | | | 0.143 | | |

R2:

Code

```
performance::r2_mckelvey(fit_alternative_base)
```

```
McKelvey's R2 
     0.159387
```

Code

```
performance::r2_mckelvey(fit_alternative_int)
```

```
McKelvey's R2 
    0.2000411
```

Simple Slope:

Code

```
interactions::sim_slopes(fit_alternative_int, pred = "Age", modx = "BMS_jn",
                         johnson_neyman = FALSE)
```

```
SIMPLE SLOPES ANALYSIS

Slope of Age when BMS_jn = 0.00 (0): 

   Est.   S.E.   z val.      p
------- ------ -------- ------
  -0.01   0.05    -0.19   0.85

Slope of Age when BMS_jn = 1.00 (1): 

   Est.   S.E.   z val.      p
------- ------ -------- ------
  -0.17   0.06    -3.04   0.00
```

Code

```
interactions::sim_slopes(fit_alternative_int, pred = "BMS_jn", modx = "Age", 
                         modx.values = c(0, .5, 1, 1.5, 2, 6),
                         johnson_neyman = TRUE, jnplot = TRUE)
```

```
JOHNSON-NEYMAN INTERVAL

When Age is OUTSIDE the interval [-33.77, 1.59], the slope of BMS_jn is p <
.05.

Note: The range of observed values of Age is [0.01, 17.73]
```

```
SIMPLE SLOPES ANALYSIS

Slope of BMS_jn when Age = 0.00: 

   Est.   S.E.   z val.      p
------- ------ -------- ------
  -0.28   0.32    -0.88   0.38

Slope of BMS_jn when Age = 0.50: 

   Est.   S.E.   z val.      p
------- ------ -------- ------
  -0.36   0.30    -1.21   0.23

Slope of BMS_jn when Age = 1.00: 

   Est.   S.E.   z val.      p
------- ------ -------- ------
  -0.44   0.28    -1.55   0.12

Slope of BMS_jn when Age = 1.50: 

   Est.   S.E.   z val.      p
------- ------ -------- ------
  -0.52   0.27    -1.91   0.06

Slope of BMS_jn when Age = 2.00: 

   Est.   S.E.   z val.      p
------- ------ -------- ------
  -0.60   0.27    -2.25   0.02

Slope of BMS_jn when Age = 6.00: 

   Est.   S.E.   z val.      p
------- ------ -------- ------
  -1.25   0.38    -3.32   0.00
```

## 6.3 Lumbar punction after event

- Model summary
- Model comparison
- R² model fit
- Alternative model approach

- Model 1: time (0 to 11) with 4-month period after meningitis event without shift.
- Model 2: time (0 to 11) with interaction, modeling shift in LP change when meningitis event occured. Interaction with time models change after event.

Code

```
fit_mod1 <- glmer(LP ~ time + TaEvent + ( 1 | year), 
                  data = df_casetotal,
                  family = "binomial")
fit_mod2 <- glmer(LP ~ time * pathogen + ( 1 | year), 
                  data = df_casetotal,
                  family = "binomial")

sjPlot::tab_model(fit_mod1, fit_mod2,
                  dv.labels = c("4 month intervall", 
                                "Change after event"),
                  show.se = TRUE)
```

|  | 4 month intervall | | | | Change after event | | | |
| --- | --- | --- | --- | --- | --- | --- | --- | --- |
| Predictors | Odds Ratios | std. Error | CI | p | Odds Ratios | std. Error | CI | p |
| (Intercept) | 0.01 | 0.00 | 0.01 – 0.01 | **<0.001** | 0.01 | 0.00 | 0.01 – 0.01 | **<0.001** |
| time | 0.98 | 0.02 | 0.95 – 1.02 | 0.347 | 1.00 | 0.02 | 0.96 – 1.04 | 0.927 |
| TaEvent | 1.19 | 0.05 | 1.08 – 1.30 | **<0.001** |  |  |  |  |
| pathogen |  |  |  |  | 1.93 | 0.67 | 0.98 – 3.79 | 0.057 |
| time × pathogen |  |  |  |  | 0.93 | 0.05 | 0.84 – 1.04 | 0.208 |
| Random Effects | | | | | | | | |
| σ2 | 3.29 | | | | 3.29 | | | |
| τ00 | 0.01 year | | | | 0.04 year | | | |
| ICC | 0.00 | | | | 0.01 | | | |
| N | 6 year | | | | 6 year | | | |
| Observations | 30967 | | | | 30967 | | | |
| Marginal R2 / Conditional R2 | 0.017 / 0.019 | | | | 0.003 / 0.016 | | | |

AIC /BIC for the first model was lower.

Code

```
anova(fit_mod1, fit_mod2)
```

```
Data: df_casetotal
Models:
fit_mod1: LP ~ time + TaEvent + (1 | year)
fit_mod2: LP ~ time * pathogen + (1 | year)
         npar    AIC    BIC  logLik -2*log(L) Chisq Df Pr(>Chisq)
fit_mod1    4 3076.8 3110.1 -1534.4    3068.8                    
fit_mod2    5 3087.6 3129.3 -1538.8    3077.6     0  1          1
```

Code

```
performance::r2_nakagawa(fit_mod1)
```

```
# R2 for Mixed Models

  Conditional R2: 0.019
     Marginal R2: 0.017
```

Code

```
performance::r2_nakagawa(fit_mod2)
```

```
# R2 for Mixed Models

  Conditional R2: 0.016
     Marginal R2: 0.003
```

McKelvey & Zavoinas R² added after peer review:

Code

```
performance::r2_mckelvey(fit_mod1)
```

```
McKelvey's R2 
   0.01995098
```

Code

```
performance::r2_mckelvey(fit_mod2)
```

```
McKelvey's R2 
   0.01308067
```

After review: model without random structure.

Code

```
.df_casetotal <- df_casetotal |> 
  mutate(year = case_when(year == 2019 ~ 1,
                          year == 2020 ~ 2,
                          year == 2021 ~ 3,
                          year == 2022 ~ 4,
                          year == 2023 ~ 5,
                          year == 2024 ~ 6)) |> 
  mutate(time_new = as.numeric(month)*year-1)

fit_alternative1 <- glm(LP ~ time_new + TaEvent, 
                            data = .df_casetotal, family = binomial)
fit_alternative2 <- glm(LP ~ time_new * pathogen, 
                           data = .df_casetotal, family = binomial)
tab_model(fit_alternative1, fit_alternative2)
```

```
Profiled confidence intervals may take longer time to compute.
  Use `ci_method="wald"` for faster computation of CIs.
Profiled confidence intervals may take longer time to compute.
  Use `ci_method="wald"` for faster computation of CIs.
```

|  |  |  |  |  |  |  |
| --- | --- | --- | --- | --- | --- | --- |
|  | LP | | | LP | | |
| Predictors | Odds Ratios | CI | p | Odds Ratios | CI | p |
| (Intercept) | 0.01 | 0.01 – 0.01 | **<0.001** | 0.01 | 0.01 – 0.01 | **<0.001** |
| time new | 1.00 | 0.99 – 1.01 | 0.999 | 1.00 | 1.00 – 1.01 | 0.377 |
| TaEvent | 1.19 | 1.10 – 1.29 | **<0.001** |  |  |  |
| pathogen |  |  |  | 1.73 | 0.87 – 3.20 | 0.100 |
| time new × pathogen |  |  |  | 1.00 | 0.98 – 1.01 | 0.621 |
| Observations | 30967 | | | 30967 | | |
| R2 Tjur | 0.001 | | | 0.000 | | |

R2:

Code

```
performance::r2_mckelvey(fit_alternative1)
```

```
McKelvey's R2 
   0.01794219
```

Code

```
performance::r2_mckelvey(fit_alternative2)
```

```
McKelvey's R2 
  0.005504486
```

# 7. Plots for publication

Code

```
.df_plot <- df_allpat |> 
  group_by(year, month) |> 
  summarise(pathogen = sum(pathogen, na.rm = TRUE),
            LP = sum(LP),
            Pat_Total = sum(Pat_Total, na.rm = TRUE),
            .groups = "drop") |> 
  mutate(year = case_when(year == "2019" ~ 0,
                          year == "2020" ~ 12,
                          year == "2021" ~ 24,
                          year == "2022" ~ 36,
                          year == "2023" ~ 48,
                          year == "2024" ~ 60)) |> 
  mutate(time = as.numeric(month)) |> 
  mutate(time = time + year) |> 
  mutate(pathogen = as.factor(pathogen))

.df_fig2 <- .df_plot |> 
  mutate(n_Pat = Pat_Total - LP) |> 
  dplyr::select(-Pat_Total) |> 
  pivot_longer(cols = c(LP, n_Pat)) |> 
  mutate(alpha = ifelse(name == "n_Pat", 0.5, 1)) |> 
  mutate(name = factor(name, levels = c("n_Pat", "LP"),
                       ordered = TRUE))

fig2 <- .df_fig2 |> 
  ggplot(aes(x = time)) +
  geom_col(data = .df_plot,
           mapping = aes(y = Pat_Total, color = pathogen), fill = "white") +
  geom_col(mapping = aes(y = value, fill = name, alpha = alpha), width = .80) +
  scale_fill_manual(values = c("steelblue", "orange")) +
  labs(
    subtitle = "<span style = 'font-size:12pt'>Amount of inpatients without and <span style = 'color:orange;'>with</span> lumbar puncture. Months with <span style = 'color:steelblue'> meningits infection events</span> are highlighted if at least one event were counted.",
    x = "Month",
    y = "Patients [n]"
  ) +
  scale_y_continuous(breaks = seq(0, 600, 50),
                     expand = expansion(mult = 0, add = c(0, 25))) +
  scale_x_continuous(limits = c(0, 73),
                     breaks = seq(1, 72, 1),
                     labels = rep(c("Jan.", "", "Mar.", "", "May", "", "Jul.",
                                    "", "Sep.", "", "Nov.", ""), 6),
                     expand = expansion(mult = 0, add = .5)) +
  annotate(x = 1, xend = 12, y = 610, yend = 610, geom = "segment") +
  annotate(label = "2019", y = 620, x = 6, geom = "text", hjust = 0) +
  annotate(x = 13, xend = 24, y = 610, yend = 610, geom = "segment") +
  annotate(label = "2020", y = 620, x = 18, geom = "text", hjust = 0) +
  annotate(x = 25, xend = 36, y = 610, yend = 610, geom = "segment") +
  annotate(label = "2021", y = 620, x = 30, geom = "text", hjust = 0) +
  annotate(x = 37, xend = 48, y = 610, yend = 610, geom = "segment") +
  annotate(label = "2022", y = 620, x = 42, geom = "text", hjust = 0) +
  annotate(x = 49, xend = 60, y = 610, yend = 610, geom = "segment") +
  annotate(label = "2023", y = 620, x = 54, geom = "text", hjust = 0) +
  annotate(x = 61, xend = 72, y = 610, yend = 610, geom = "segment") +
  annotate(label = "2024", y = 620, x = 66, geom = "text", hjust = 0) +
  scale_color_manual(values = c("white", "steelblue")) +
  theme_classic() +
  theme(panel.spacing = unit(0, "pt"),
        legend.position = "none",
        plot.subtitle = element_markdown(),
        axis.title = element_text(face = "bold")
  )

# activate export if needed
#tiff(filename = "figure2.tiff", width = 15, height = 8, units = "in", pointsize = 12, res = 100)
fig2
```

Code

```
#dev.off()
```
